# Individual differences in sensitivity to positive home environment among children “at risk” for attention-deficit/hyperactivity disorder: A review

**DOI:** 10.3389/fpsyt.2022.927411

**Published:** 2022-07-22

**Authors:** Tzlil Einziger, Andrea Berger

**Affiliations:** ^1^Department of Behavioral Sciences, Ruppin Academic Center, Emek Hefer, Israel; ^2^Department of Psychology, Ben-Gurion University of the Negev, Beer Sheva, Israel; ^3^Zlotowski Center for Neuroscience, Ben-Gurion University of the Negev, Beer Sheva, Israel

**Keywords:** ADHD, sensitivity to the environment, differential susceptibility, vantage sensitivity, executive functions

## Abstract

Although the evidence for the genetic basis of attention-deficit/hyperactivity disorder (ADHD) is strong, environmental factors, such as the quality of parenting or the home environment, may moderate such genetic liability. The plausible negative effect of a low-quality home environment and negative parenting on child outcomes is well-established; however, the positive effect of a high-quality environment and positive parenting remained largely uninvestigated. Due to the presence of genetic, temperamental, or physiological factors, children who were traditionally considered *at-risk* for ADHD may be more sensitive to aspects of their environment compared to children who are not at such *risk*. Therefore, they would be more affected by their environmental experience, either for good or bad. Under supportive environmental conditions, such *at-risk* individuals might actually outperform their non-vulnerable peers, suggesting that these individual factors might be considered susceptibility factors rather than risk factors. Little is known regarding the positive effect of the environment in the ADHD literature, but it has been demonstrated in cognitive functions that are closely associated with ADHD, such as executive functions (EF). We review this literature and examine the extant empirical support for sensitivity to aspects of the home environment and parenting in the case of ADHD and EF. Moreover, we review factors that could help identify the specific aspects of the home environment and parenting that these children might be more susceptible to. Such knowledge could be valuable when designing preventive interventions and identifying those children that are especially sensitive and could benefit from such interventions. Recommendations for future studies are discussed as well.

## Introduction

Attention-deficit/hyperactivity disorder (ADHD) is a chronic neurodevelopmental disorder, among the most commonly diagnosed disorders during childhood, with an estimated worldwide prevalence of 5.9% of school-age children (1, 2) and about 2.8% of adults (3, 4). The etiology of ADHD is complex; it involves multiple factors (5, 6) that can be represented on a risk-protection continuum (7, 8) and arise from the individual himself (e.g., genetic and temperamental tendencies), the family (e.g., aspects of parenting and the home environment), and the community (7).

The heritability of ADHD was estimated to be as high as 70–80%, and it is considered to have a substantial genetic component (5, 9). Despite such high heritability, not all children who are at genetic risk for ADHD will eventually manifest the disorder’s symptoms. There is an important role of the environment, which is reflected in its plausible ability to strengthen or attenuate the genetic predisposition of ADHD (10, 11). It has been suggested that some of the genetic/temperamental risk factors of ADHD could actually reflect individual differences in sensitivity to the environment, meaning that some individuals are more sensitive to their home environment than others, and that this sensitivity can be related to their developmental outcomes (12–15). For example, individuals at such *risk*, who also experience a low-quality home environment, would be at higher risk to manifest the disorder’s symptoms, compared to individuals who are not at such *risk* (14, 16). However, individuals at *risk* who experience a very supportive environment might disproportionally benefit from this experience, and even, for certain developmental outcomes, outperform their peers who are not at such *risk* (12, 17–22). Therefore, these individual factors have been suggested to be *susceptibility* factors, rather than simply *risk* factors (23).

Understanding how sensitivity to the environment might influence the development of children is very important, especially in those cases when children could be predisposed to maladaptive outcomes in the future, such as ADHD and other developmental difficulties. In this review, we discuss the plausible role of sensitivity to the environment in the developmental pathways leading to ADHD. Specifically, we focus on the potentially positive role of parenting and home environment to shift developmental pathways of children who are *at-risk* for ADHD toward more adaptive development outcomes.

We refer to the sources of risk for ADHD as follows: a. a child’s individual factors (including genetic and temperamental tendencies), and b. environmental factors (including parenting and home environment), for which the involvement in ADHD might be moderated by the child’s individual factors. Following the literature, we also use the term “familial risk for ADHD,” which refers to the existence of parental ADHD symptoms, which might increase the risk both *via* the genetic heritage and *via* the family home environment (16).

This review consists of four sections. In Section “Introduction,” we discuss the general idea of sensitivity to the environment and review empirical evidence supporting this idea regarding general developmental outcomes. In Section “Sensitivity to environmental factors in attention-deficit/hyperactivity disorder,” we discuss sensitivity to the environment in the case of ADHD. Specifically, we review individual factors (i.e., genetic and temperamental factors) that were found to be related to ADHD and have been traditionally referred to as risk factors for ADHD. We then review evidence suggesting their role as susceptibility factors that moderate the effects of the environment on ADHD symptomatology. In Section “The role of sensitivity to the environment in the development of executive functions,” we discuss the possibility that the relation between *risk* factors for ADHD and the phenotypic manifestation of ADHD symptoms might be indirect, through the development of deficits in cognitive domains. We focus on deficits in EF as an example of such an indirect relation (although deficits in other domains might also be involved in the developmental pathways of ADHD). Finally, in Section “Discussion,” we discuss the general implications of sensitivity to the environment in the context of ADHD, the current limitations in the extant research in this field, and discuss future research directions.

### Individual differences in sensitivity to the environment

The general idea that children vary in their sensitivity to environmental settings or parenting practices is a cornerstone in the field of developmental psychology. This idea has been largely supported by empirical evidence that repeatedly demonstrated interactions between child characteristics and their rearing environment, in the prediction of a wide range of developmental outcomes, such as externalizing behaviors, substance abuse, internalizing problems, disorganized attachment, prosocial behaviors, self-regulation, and more (24–32).

Interactions between child characteristics and aspects of the rearing environment have been mainly interpreted according to several theoretical frameworks, including the diathesis-stress model (33), the differential susceptibility model (23), and the vantage sensitivity model (34). The basic assumption underlying all these models is that some individuals are more sensitive to environmental input than others; this sensitivity is based on genetic, temperamental, or physiological factors (23, 33). The difference between these models lies in the specific range that each model refers to on the scale from a poor rearing environment to a supportive and enriching environment.

According to the diathesis-stress model (33), some individuals are more vulnerable to risks in their environment, such as harsh circumstances, poor-quality home environment, and parenting. The vulnerability factor (i.e., diathesis) is not expressed by itself; rather, it is expressed in combination with poor environmental experience (i.e., stress) and creates a “double risk” for the child to develop a maladaptive outcome, such as psychopathology.

However, an environment can also be positive and supportive. The differential susceptibility model (23) proposes that individual differences can also be found in the sensitivity to plausible positive environmental influences. Therefore, according to this framework, risk or vulnerability factors can be referred to as susceptibility factors. Susceptible individuals would be more affected by negative environmental experiences (similar to the prediction of the diathesis-stress model) but also benefit disproportionally from positive environmental experiences, compared to their non-susceptible peers. Namely, the same characteristics that make them vulnerable to poor environmental experience, also make them benefit from a positive environmental experience; therefore, under a supportive environment, they would outperform their non-susceptible peers. Thus, such susceptibility factors make them more sensitive to their environment, “for better and for worse” (23, 26, 27).

**FIGURE 1:**
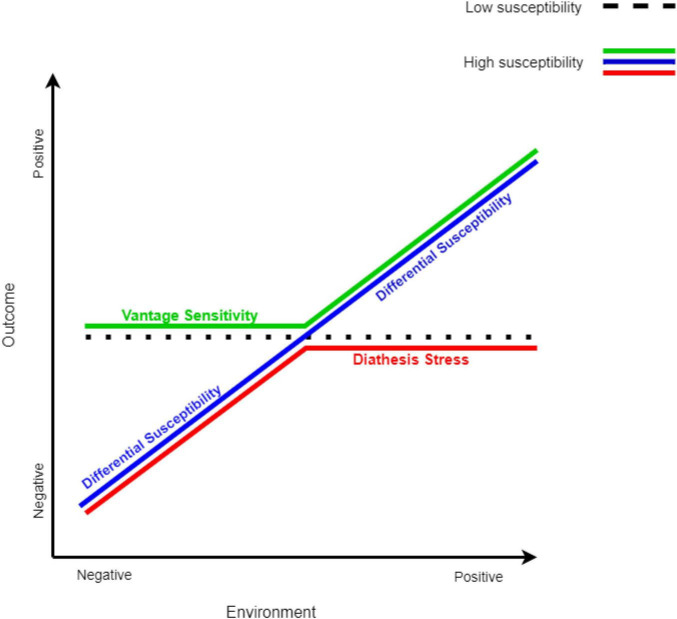
Models of sensitivity to the environment. The models of sensitivity to the environment are adapted from Bakermans-Kranenburg and van IJzendoorn (155). All three models predict that non-susceptible individuals are not affected by the environment (dotted black line). The red line represents susceptible individuals according to the diathesis-stress model, the green line represents susceptible individuals according to the vantage sensitivity model, and the blue line represents susceptible individuals according to the differential susceptibility model.

Following the differential susceptibility model, the vantage sensitivity model (34) was developed to describe the tendency of some individuals to benefit more than others from positive environmental experiences. According to this model, some individuals may be more susceptible to enrichment and support in their environment, but they do not show such sensitivity to the negative aspects of their environment. This means that these individuals are more responsive to environmental enrichment, but they are also resilient and protected from adversity (34). An illustration of the three theoretical models is presented in Figure 1.

Pluess (35) suggested an integrated model, in which the different types of sensitivity to the environment can be viewed as part of a single framework. Evidence that shows that the same individual’s factors (e.g., gene variations) predict both sensitivities to negative and positive aspects of the environment, may imply that the different models reflect the same propensity for sensitivity to the environment. However, the difference between these types of sensitivities (i.e., to a negative environment, a positive environment, or both) is shaped both by the presence of susceptibility factors and by early life experiences (36, 37). According to this view, individuals who display a predisposition for sensitivity to the environment may eventually develop a biased sensitivity toward negative experience (i.e., diathesis-stress) or support (i.e., vantage sensitivity), according to their early development experiences. In this sense, in an early adverse environment, this sensitivity would develop into a biased sensitivity toward negative environmental aspects (i.e., diathesis-stress); in a supportive environment, this sensitivity would develop into a biased sensitivity toward positive environmental aspects (i.e., vantage sensitivity); and in an early neutral environment, this sensitivity would develop into a sensitivity to both negative and positive environmental aspects (i.e., differential susceptibility) [for an elaboration of this integrated model, see (35)].

From an empirical point of view, one of the main difficulties in distinguishing between these models is that their empirical testing is heavily dependent on the specific range of the environmental quality that was considered (30, 34), as not all environmental aspects range from the negative to the positive side (34). For example, some negative environmental experiences range from adversity (e.g., poverty or low family income) to the absence of adversity (19, 38); however, the absence of adversity does not necessarily mean a supportive and enriching environment. Another example is inconsistent parenting, an aspect of parenting that has been studied in relation to ADHD (14). While it is acceptable that inconsistent parenting lies on the negative side of the parenting practice range, it is much less clear that consistent parenting lies on the positive range (as it can be consistent, but still negative). Likewise, positive environmental experiences often range from the positive to the absence of the positive, for example, psychological intervention vs. no intervention (34), but the absence of the positive factor does not necessarily mean a poor-quality environment.

As mentioned above, in this review, we focus on the role of sensitivity to parenting and aspects of the home environment in the development of ADHD. Sensitivity to negative aspects of the home environment, as reflected in the diathesis-stress model, has already been reviewed elsewhere (39) and therefore will only be briefly discussed here. We focus on studies that tested the positive role of the home environment or parenting on the development of ADHD and used rather a full range of the environment (i.e., negative to positive) or focused on the positive side of the range. Therefore, the review mainly discusses the differential susceptibility and vantage sensitivity models.

### Empirical evidence for sensitivity to environmental factors on general developmental outcomes

Despite the range limitations described above, several meta-analyses included studies that used varied susceptibility factors (i.e., one or more genes, different aspects of temperament), a variety of environmental aspects (that together reflect a full range from negative to positive environments), and a large range of outcomes, supported the idea of sensitivity to the environment, and specifically suggested a pattern of differential susceptibility; meaning that sensitivity to the environment was demonstrated for better and for worse (25, 30, 31). For example, two meta-analyses on gene-by-environment interactions demonstrated how the environmental experience was moderated by the 5-HTTLPR gene (31) and by dopamine-related genes (i.e., DRD2, DRD4, DAT) (24), in predicting several developmental outcomes (e.g., externalizing behaviors, disorganized attachment, prosocial behaviors, etc.). Children with the short allele of the 5-HTTLPR and children with less efficient dopamine-related genes (i.e., both suggested as susceptibility genes) had negative outcomes under negative environments, but they also profited under positive environments (25, 31). Similarly, Slagt et al.’s meta-analysis (which included 84 longitudinal studies, involving more than 6,000 children up to 18 years old) showed that children with more difficult temperaments were more sensitive to both negative and positive aspects of parenting, which was expressed in externalizing and internalizing problems as well as social and cognitive competence (30).

More recent findings also supported sensitivity to the environment in predicting internalizing and externalizing problems (32, 40, 41), and suggested sensitivity only to negative aspects of the environment (40, 41) or sensitivity to both negative and positive aspects of the environment (32). For example, in Jaekel et al.’s study, individuals with early regulation problems (i.e., persistent crying, feeding, and sleeping problems) in infancy were more sensitive to insensitive parenting (measured at age 6 years), which was reflected in attention regulation at age 8 years and internalizing behaviors at age 28 years. Such sensitivity was not found for the positive range of maternal sensitivity, and therefore, results supported a diathesis-stress model (40). In contrast, in Windhorst et al.’s study, a pattern of differential susceptibility was found when the effect of early maternal sensitivity (measure at 14 months of age) on later externalizing behavior was found, for better and for worse, among children carrying the 7-repeat allele of the DRD4 (32).

Such inconsistency in the pattern of sensitivity to the environment between the results of Jaekel et al. (40) and Windhorst et al. (32) may be explained by the age of assessment (i.e., in this case, maternal sensitivity that was assessed at 6 years and 14 months, respectively). It has been suggested that susceptibility to the environment is mainly evident early in childhood, and less so in later developmental periods (30, 42). Indeed, in a study that tested interactions between aspects of parenting and personality traits among adolescents, no evidence was found for sensitivity to the environment during adolescence (42). Also, in Slagt et al.’s meta-analysis, sensitivity to the environment was only found when child temperament (i.e., negative emotionality) was assessed during infancy; possibly because of the rapid development in this developmental period (30).

## Sensitivity to environmental factors in attention-deficit/hyperactivity disorder

### Why is it important to study sensitivity to the environment in the case of attention-deficit/hyperactivity disorder?

As detailed above, the concept of sensitivity to the environment has been well-demonstrated in relation to several aspects of child adjustment, including externalizing and internalizing disorders (25, 30, 31, 40, 41). However, ADHD has unique behavioral and cognitive aspects that may differentiate them from these disorders, and the role of sensitivity to the environment in the developmental pathways of ADHD is not fully known yet.

ADHD is one of the most common behavioral disorders in childhood (1), and it has high persistence rates throughout the life span (43–45). It is related to multiple negative long-term outcomes; for example, greater risk has been reported for poor academic performance (46, 47), difficulties in employment (48), and several psychosocial problems, such as poor social skills, low self-esteem, and emotional dysregulation (40, 49–53). It is also associated with risk-taking behaviors in several life domains (54), such as substance abuse (55, 56), criminal behavior (57, 58), driving risks (59), and gambling (60).

Individuals with ADHD are also at risk for secondary mental health problems; indeed, comorbidities with other psychopathologies are frequent, especially externalization problems such as oppositional defiant disorder (ODD) and conduct disorder, internalizing problems such as mood and anxiety disorders, and specific learning disorders (44, 45, 61, 62). Researchers have suggested that some of these serious conditions, such as depression and substance abuse, are causally explained by an earlier onset of ADHD (63, 64). From an economic perspective, and beyond the substantial personal suffering, ADHD has also significant societal costs which burden healthcare services (65, 66).

All these aspects emphasize the importance of early detection of children at risk to develop ADHD and early implementation of intervention programs. Potentially, such programs could moderate the developmental course of ADHD and lead to a decrease in the likelihood of ADHD diagnosis and persistence rates, as well as diminishing or even preventing long-term outcomes that are associated with ADHD. An early preventive approach is important because serious difficulties and negative outcomes may persist despite the decline of symptomatology in adolescence and adulthood (67). The design of such intervention programs requires a vast understanding of the protective factors that may increase the child’s resilience against his initial familial/genetic risk. Therefore, the idea of sensitivity to the environment may have important implications for the implementation of early interventions. Understanding which children may show increased sensitivity to which specific home environmental or parenting aspect could help in tailoring the intervention to aspects that the child may particularly benefit from. Interventions might be most beneficial when implemented in early childhood because brain plasticity may be greatest during the early phases of development (68, 69); therefore, environmental sensitivity may be enhanced among young children. Furthermore, intervention can be implemented before any behavioral patterns become strong habits and before negative experiences associated with school failure occur (70).

### “Risk” or susceptibility factors of attention-deficit/hyperactivity disorder

In this section, we discuss individual factors (i.e., genetic and temperamental factors) that have been traditionally referred to as risk factors for ADHD (71, 72); evidence for their potential role as susceptibility factors in the development of ADHD is discussed in section “Empirical evidence for sensitivity to the environment in attention-deficit/hyperactivity disorder.”

Multiple genetic factors and their interactions with environmental factors play a substantial role in the etiology of ADHD (5, 6, 73). Early molecular genetic studies have mainly focused on genes in catecholaminergic pathways because this is the target of the most effective drug treatments for the disorder (methylphenidate and amphetamine) (71). These studies examined the DNA variants of candidate genes and found evidence for a small number of potential genes that have been consistently associated with ADHD across studies, such as the D4 dopamine receptor gene (DRD4), the dopamine transporter gene (DAT1), and the serotonin transporter gene (5HTTLPR) (71, 72, 74, 75). Specifically, the DRD4 polymorphism has a 48-bp repeat sequence in exon III. The 2-, 4-, and 7-repeat alleles are the most frequent variation, and the 7-repeat allele (7-DRD4) has been specifically suggested to be a marker of risk for ADHD (71, 76). The DAT1 polymorphism has a 40 bp repeat located in the 3” untranslated region of the gene. The two most prevalent allelic variants are the 9-repeat and the 10-repeat. The 10-repeat variation has consistently been found to be associated with ADHD (71, 74, 77). The 5HTTLPR is a degenerate repeat polymorphic region in the SLC6A4, it comprises a short (s) and a long (l) allele. Children carrying the short allele, and particularly those who are homozygous for this allele (s/s), are considered at risk for ADHD (78, 79). However, this genetic risk tended to have small effect sizes in predicting ADHD, and it was also associated with increased risk for other psychopathologies and therefore not specific to ADHD (72, 75).

A more advanced approach was used by genome-wide association studies, which scan the entire genome. Such studies showed that the genetic risk for ADHD is due to the polygenic effects of numerous common and rare DNA variants. Each variant explained a very small proportion of the variance of ADHD and their combination increases the risk for ADHD; together these variants explained approximately one-third of ADHD heritability (6, 80). The polygenic risk for ADHD was found to be related to sub-threshold levels of ADHD in the general population (80, 81), but also to other psychiatric disorders (82).

Another individual risk factor that has been studied in relation to ADHD is child temperament. Temperament refers to innate individual differences in behavioral tendencies and style, which appear early in life and remain relatively stable during development (83, 84); it is considered to be the core root for the development of a child’s personality (85). According to Rothbart’s conceptualization, temperament is defined as constitutionally based individual differences in two higher-order domains: reactivity and self-regulation (84). The reactivity domain emerges as early as the first months of life and refers to levels of bottom-up responsivity to changes in the environment and levels of arousal in affect, motor, and automatic responses (including aspects such as activity level, impulsivity, negative affectivity, and sociability). The self-regulation domain, also referred to as effortful control (EC), emerges later in childhood and includes more deliberate top-down processes that can affect and moderate the reactivity domain; it depends on the development of executive aspects of attention and includes aspects of attentional focusing, attentional shifting, and inhibitory control (84–87).

Researchers have suggested that at extreme levels, certain temperament dimensions may act as markers of risk for ADHD (88–90). In general, aspects of higher reactivity and lower self-regulation are related to ADHD symptomatology among children (88, 91–98). Specifically, children with ADHD symptomatology demonstrated higher levels of negative affectivity (especially anger) than control participants (91, 95, 99), higher levels of surgency and especially motor activity (90–92, 100, 101), and lower levels of EC (91–95, 101, 102).

Importantly, genetic risk for ADHD and aspects of child temperament are not exclusive risk factors. For example, Martel et al.’s have recently found a mediational pathway from polygenic risk for ADHD, through temperamental EC, to inattention symptoms among adolescents (103). However, in most cases, studies use either genetic risk factors or temperamental risk factors; therefore, these factors were studied as separate markers of sensitivity to the environment.

### Empirical evidence for sensitivity to the environment in attention-deficit/hyperactivity disorder

Environmental factors were estimated to account for 10–40% of the variance of ADHD through their direct effect or gene-environment interactions (104). The environmental factors that were found to be associated with ADHD include, for example, prenatal factors, pregnancy and delivery complications, low birth weight, exposure to toxins (e.g., lead), and aspects of the home environment, such as low socioeconomic status (SES) and psychological adversity (104–106). Environmental factors may modify the expression of the genetic/biological liability on child outcomes and bring the disorder to fruition (10). A review on G × E interactions in ADHD suggested that evidence for such interactions, involving psychosocial factors such as aspects of the home environment, were more replicable compared to interactions involving prenatal and perinatal factors (107). Indeed, aspects of parenting and home environment may have an important role in shaping developmental pathways leading to the onset, persistence, or decline of ADHD symptoms throughout development (11); and these aspects can be changed and modified as part of an intervention program such as parent training.

It should also be considered that child ADHD may be bi-directionally related to the family environment (108–110). For example, in a longitudinal cross-lagged design, greater child ADHD symptoms were found to predict greater maternal life stress, maternal depressive symptoms, and lower warmth; and greater maternal life stress and overreactive parenting predicted greater child symptoms (108). Indeed, there is evidence to support both child effects on parenting (109–112) and the parenting/home environment effect on the child (113–116). Therefore, early detection of susceptible children and targeting specific aspects of parenting and home environment, that these children may be especially sensitive to, may help to prevent the negative cycle that child behavior adversely affects the parents, and the parents’ behavior negatively affects the child.

The existence of sensitivity to aspects of parenting and home environment in ADHD has been supported by several studies including studies that used the candidate genes approach (12–14, 117–120), a few studies that examined temperament-by-parenting interactions (15, 22, 96, 101), and a single study that examined the familial risk for ADHD according to maternal ADHD symptoms (16). In the next sections, we briefly describe more traditional evidence that supported a sensitivity to negative aspects of the home environment and then discuss more recent evidence that examined the plausible positive effect of parenting and the home environment.

#### Sensitivity to negative aspects of the home environment

The negative effect of aspects of the parenting and home environment on children at risk for ADHD has been supported by several studies that used environmental aspects such as low SES, low parental education, and psychological adversity, supporting a diathesis-stress model (39, 118–121). A detailed review of these studies can be found in Pennington et al.’s review (39), which focused on the diathesis-stress effect in ADHD. Evidence for the diathesis-stress model was also found in more recent studies that were not included in Pennington’s review (14, 16, 122). For example, Martel et al. found that susceptible children (children homozygous for the 7-repeat allele of the DRD4 gene) showed a higher rating of ADHD and ODD symptoms, but only in the presence of inconsistent parenting. Under consistent parenting, susceptible children did not differ from non-susceptible children; their results supported a diathesis-stress model (14). Another support for a diathesis-stress model was found in Auerbach et al.’s longitudinal study. They found that familial risk for ADHD (according to maternal hyperactivity/impulsivity symptoms) predicted higher levels of child hyperactivity/impulsivity symptoms in elementary school age, but only in a negative home atmosphere (that was characterized by high levels of conflict and chaos). In a less negative environment, this familial risk did not predict child symptoms (16).

Although all the findings in this section supported the diathesis-stress model (14, 16, 118–122), it should be noted that the range of environmental variables did not cover the full range from negative to positive experience, but rather from negative to perhaps neutral, or less negative environment; for example, aspects of adversity vs. the absence of adversity (118–122), inconsistent vs. consistent parenting (14) and negative home atmosphere vs. less negative home atmosphere (16). As these studies did not necessarily include a more positive and enriching side of parenting or the home environment, the plausible positive role of the environment remained unclear. Still, despite the restricted environmental range, these findings support the general idea of sensitivity to the environment in ADHD.

#### Sensitivity to positive aspects of the home environment

Much less is known regarding such sensitivity to positive aspects of the environment in the case of ADHD. In this section, we discuss evidence from studies that tested a broader range of the home environment or tested the plausible role of positive parenting or home environment on the development of ADHD. An overview of these studies can be found in Table 1.

**TABLE 1:** An overview of studies that tested sensitivity to positive aspects of the environment in ADHD.

Study	Age of participants	Susceptibility factor	Environmental factor	Range of environmental factor	Outcome	Theoretical model that was supported	Comments
Li and Lee (123)	6–9 years	MAO-A genotype	Negative and positive parenting	Full range (negative to positive)	ADHD symptoms	Negative parenting by genotype interaction was found, but the specific pattern was not tested	Results were specific to inattention symptoms
Li and Lee (117)	6–9 years	DAT1 genotype	Observed parenting behavior (negativity and praise)	Full range (negative to positive)	ADHD symptoms	Significant interactions were found between genotype and parenting, but neither model was fully supported	
Elmore et al. (13)	6–17 years	5-HTTLPR	Family conflict and cohesion	Full range (negative to positive)	ADHD symptoms	Differential susceptibility	Results were specific to inattention symptoms
Baptista et al. (12)	36–77 months	5-HTTLPR	Caregiver intrusiveness	Only negative range	ADHD symptoms	Differential susceptibility	
Xing et al. (22)	14–22 months	Temperamental reactivity	Mother personality (and specifically conscientiousness)	Full range (negative to positive)	Impulsivity	Differential susceptibility	ADHD symptoms were not fully assessed
Miller et al. (96)	7–9 years	Motor activity and positive affect	Maternal caregiving behaviors	Full range (negative to positive)	ADHD symptoms	A parenting by temperament interaction was found, but the specific pattern was not tested	
Rioux et al. (15)	4–7 years	Inhibitory control	Positive parenting	Only positive range	ADHD symptoms	Vantage sensitivity	
Hare and Graziano (101)	5 years	Effortful control	Inconsistent discipline	Only negative range	ADHD symptoms (pre-treatment)	Vantage sensitivity	Results were specific to hyperactivity-impulsivity

Elmore et al.’s found that the 5HTTLPR genotype moderated the relation between positive and negative aspects of the home environment (i.e., cohesion/conflict, respectively) and inattention symptoms. Specifically, susceptible youth (i.e., s/s homozygotes) who experienced high cohesion showed decreased inattention symptoms, compared to non-susceptible youth; but, susceptible youth who experienced high conflict showed increased inattention symptoms, supporting a differential susceptibility pattern (13).

In a study that was conducted on a sample of preschoolers who were not raised by their biological parents, consistent findings regarding the positive effects of caregiving on the development of ADHD symptoms in children emerged. Susceptible children according to their 5HTTLPR genotype (i.e., s/s homozygotes) who experienced less intrusive caregiving demonstrated fewer ADHD symptoms, even after controlling for age at placement or duration of institutionalization, but those susceptible children who experienced more intrusive caregiving demonstrated the highest ADHD symptoms. This pattern was consistent with a differential susceptibility model (12), although it should be noted that the environmental measure did not cover a full range of negative to positive environments (i.e., less intrusive caregiving does not necessarily reflect positive parenting).

General support for sensitivity to the environment was also found in Li and Lee’s study; observed parenting behaviors, and specifically praises (an aspect of positive parenting), were associated with increased ADHD only among children who carried the 9/10 allele of the DAT1. Negative parenting behaviors were positively associated with ADHD only among children who carried the 9/9 allele (117). These results suggest that different children show differences in their sensitivity to aspects of positive and negative parenting, but they are not in line with either of the mentioned theoretical models (i.e., diathesis-stress, differential susceptibility, vantage sensitivity). Furthermore, they do not show that susceptible children may benefit from the positive environmental experience, but rather children who were exposed to positive aspects of parenting (i.e., praise) showed increased symptomatology. In another study by Li and Lee, susceptibility was measured by the monoamine oxidase A (MAO-A) genotype.^1^ Li and Lee used a sample of 6–9 year-old-boys and found that negative aspects of parenting predicted child inattention symptoms, but only among boys with high-activity MAO-A genotype, supporting sensitivity to negative aspects of the environment. However, this genotype did not moderate the association between positive parenting and child symptomatology (123). Therefore, no evidence for sensitivity to positive aspects of parenting was found.

Aspects of temperamental reactivity (22, 96), inhibitory control (15), and effortful control (101) were also found to indicate susceptibility to the environment. In Xing et al.’s study, infant temperamental reactivity was found to moderate the effect of maternal conscientiousness on infants’ impulsivity.^2^ Specifically, high reactive children whose mothers had high levels of conscientiousness showed the lowest levels of impulsivity, even compared to low reactive children. However, high reactive children whose mothers had lower conscientiousness showed the highest levels of impulsivity, suggesting a pattern of differential susceptibility (22).

In Miller et al.’s longitudinal study, an interesting sex-specific pattern of temperament-by-parenting interaction was found. For girls, temperamental aspects of positive affect and motor activity, measured at 4 months of age, longitudinally predicted lower ADHD symptoms at 7 and 9 years, but only among those who experienced a high quality of maternal caregiving behaviors. For girls who experienced lower quality maternal caregiving behaviors, positive affect predicted higher ADHD symptoms. This may indicate a sensitivity to both positive and negative aspects of parenting. For boys, infant motor activity at 4 months of age predicted higher ADHD symptoms at 7 and 9 years, but only among those who experienced lower quality of maternal caregiving behaviors, which may indicate such sensitivity, but only to negative aspects of parenting (96). It should be noted that this study did not include a direct statistical test of diathesis-stress or differential susceptibility; however, the moderating role of maternal caregiving behaviors on the longitudinal relation between temperamental factors and the later manifestation of symptoms of ADHD may still indicate sensitivity to the environment among temperamentally susceptible children. It also emphasizes the importance of testing gender-specificity in such interactions.

Another evidence of sensitivity to the environment was found in the study of Rioux et al.; positive parenting practice predicted lower levels of ADHD symptoms at 7 years of age, but only among children with high inhibitory control. Children with lower levels of inhibitory control showed higher levels of ADHD symptoms, regardless of the level of positive parenting. These results supported a vantage sensitivity pattern because some children showed increased sensitivity to positive aspects of parenting exclusively (15). A similar pattern of vantage sensitivity was also found in Hare and Graziano’s study, that is, an interaction between effortful control and inconsistent discipline was found; children with high effortful control who also experienced low levels of inconsistent discipline, showed lower levels of hyperactivity-impulsivity, meaning that the protective effect of high effortful control only occurred when combined with this environmental aspect (101), although, as discussed, this does not necessarily reflect a positive environment.

It should be noticed that in both the study of Rioux et al. (15) and of Hare and Graziano (101), sensitivity to the environment was found among those who are considered at lower *risk* for ADHD (i.e., high on inhibitory control/effortful control); whereas children at high *risk* are those who are usually found to be more susceptible to their environment. However, temperamental susceptibility in both studies was based only on one domain, and since not all individuals with ADHD show deficits in this area (124), it is unclear whether this high inhibitory control/effortful control group was more sensitive to their environment due to other temperamental aspects. It still raises an interesting possibility that for some environmental aspects, children who seem to be more resilient may be those who benefit more from a positive environment.

### Interim summary

To summarize so far, there are some empirical indications of sensitivity to the environment in the case of ADHD, but the specific pattern of interaction was found to vary between the studies that looked at different environmental aspects. Some studies found evidence for a diathesis-stress pattern (14, 16, 39, 118, 120, 122); meaning that susceptible individuals were only sensitive to the negative aspects of the environment. Although, as noted, most of these studies focused on a negative-neutral range of the environmental aspect and did not include aspects of positive parenting or home environment. Other studies found evidence of differential susceptibility in predicting ADHD symptomatology, meaning that susceptible individuals were more sensitive to both negative and positive aspects of the environment (12, 13); such a pattern was also found in a study that examined impulsivity behaviors, which are considered a main symptom domain of ADHD, but ADHD symptoms were not fully assessed (22). Other studies found evidence of a vantage sensitivity pattern (15, 101), meaning that some individuals were sensitive only to positive, or less negative, aspects of the environment. Two studies found some indications of sensitivity to the environment, although their results did not entirely fit any of the patterns (117), or the specific pattern was not directly tested (96). One study that tested the effect of positive and negative parenting found evidence of sensitivity to the environment, but only to negative aspects of parenting (123).

Although all these studies have directly tested the outcome of ADHD symptoms [besides one study that focused solely on impulsivity (22)], it is important to consider that the behavioral manifestation of ADHD is very heterogenic, not only in terms of the specific expression of ADHD symptom domains (i.e., inattention and hyperactivity/impulsivity), associated comorbid problems, and long-term outcomes (10, 125, 126), but also in the level of cognitive deficits (127). Not all cognitive deficits are exhibited by all individuals with ADHD (128); different deficits are assumed to be involved in distinct developmental pathways leading to ADHD (129). Compared to the behavioral manifestation of ADHD symptoms, these cognitive deficits are assumed to be genetically less complex and therefore may be more strongly related to the disorder’s etiological factors (10, 128, 130, 131).

Furthermore, examining the outcome of ADHD symptoms may not be ideal for fully understanding sensitivity to the environment because a negative developmental outcome of ADHD symptomatology is usually examined against children with low symptomatology, but the lack of symptoms does not necessarily imply a positive developmental outcome. Therefore, there is some sense in examining the effect of sensitivity to the environment also regarding cognitive functioning known to be involved in ADHD and that covers the full range from negative to positive outcomes. For example, deficits in aspects of executive functions (EF) have been extensively demonstrated in a subgroup of individuals with ADHD (132–134). EF is a multidimensional construct, which includes core neurocognitive processes, such as working memory, inhibition, attentional shifting, and planning (135, 136). Deficits in aspects of EF have been consistently demonstrated in individuals with ADHD, in both clinical and community samples (132–134). Deficits in EF are considered a liability for the underlined pathology of ADHD (128, 130, 137). Such a cognitive assessment of EF may more broadly cover the range from a negative development outcome (i.e., low cognitive functioning) to a positive development outcome (i.e., high cognitive functioning).

Therefore, it should be considered that there could be indirect paths, from the initial *risk* for ADHD, through the development of deficits in cognitive domains, and then to the phenotypic manifestation of ADHD symptoms. For these reasons, it is important to examine the plausible role of sensitivity to positive aspects of the environment regarding cognitive outcomes, that may play a role in the development of ADHD, for example, aspects of EF. Testing the involvement of intermediates may also expand our understanding of how genetic or temperamental sensitivity works (138). In the next section, we briefly review evidence regarding sensitivity to the environment in the case of EF. As mentioned, we focused on deficits in EF to demonstrate the possibility of such an indirect developmental pathway. However, other cognitive and motivational factors might also play an intermediate role in the development of ADHD.

## The role of sensitivity to the environment in the development of executive functions

**FIGURE 2:**
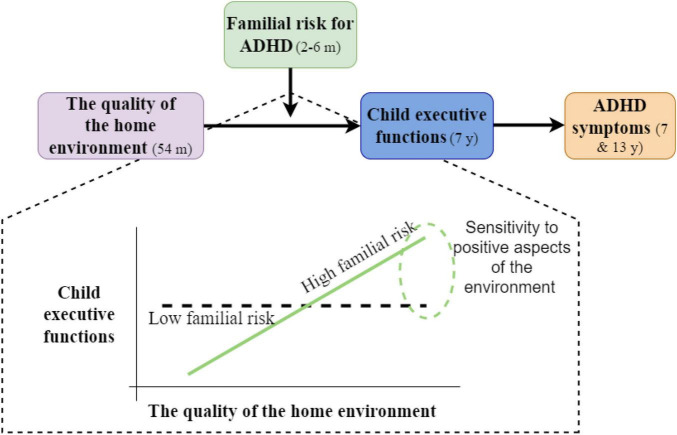
Schematic description of the findings in Einziger et al. (17).

An indirect relation between the initial familial risk for ADHD, through child EF, and the later manifestation of ADHD symptoms was recently demonstrated in Einziger et al.’s study (17). In this study, child familial risk was assessed by parental ADHD symptomatology, which was measured 2–6 months after their child’s birth. Such risk is not entirely genetic because it might be the result of aspects of parenting and the home environment. Indeed, adults with ADHD show deficits in aspects of EF and emotional regulation (125, 139), which might interfere with the necessary skills for effective parenting, such as parenting control behaviors and emotional responsiveness (140–142). Einziger et al. used a longitudinal design that followed children from birth to adolescence, and found that familial risk for ADHD, based on both parents’ ADHD symptomatology, interacted with the quality of the home environment in early childhood in predicting child cognitive functioning at elementary school age; this cognitive functioning, in turn, predicted adolescents ADHD symptomatology. Specifically, it was found that in a lower-quality home environment, children at high familial risk showed poor cognitive functioning (including low levels of EF) at elementary school age, but under a very supportive environment, they showed high cognitive functioning (including high levels of EF), outperforming their peers who were at low familial risk. This pattern of results suggested that such familial risk may act as a susceptibility factor, rather than a risk factor. When testing more specific domains of the home environment, a similar pattern of results was found for aspects of cognitive stimulation (i.e., an environment that is rich in cognitive stimulation includes the presence of adequate learning and language materials, and parental involvement to encourage the child to engage in enriching activities). However, the effect was not found for aspects of emotional support (e.g., warmth, acceptance of the child, etc.), which suggested that this sensitivity to the environment may be domain-specific. Furthermore, child EF was involved in a developmental pathway leading to ADHD symptoms in elementary school and adolescence; it mediated the relation between the early risk for ADHD and the later manifestation of ADHD symptomatology (17). These findings suggest that sensitivity to the home environment in early childhood may be related to the later cognitive functioning of children; for children at familial risk for ADHD, a supportive home environment may be related not only to less negative outcomes but also to positive cognitive functioning, which may be then translated to lower ADHD symptomatology. See Figure 2 for a schematic description of these findings.

There are some additional studies showing evidence regarding sensitivity to the environment in the development of aspects of EF (18–21, 38, 143, 144), although these studies did not examine the full path up to the emergence of ADHD symptoms. For example, Vrantsidis et al. found evidence for a diathesis-stress pattern because children who carried susceptibility alleles (i.e., COMT, DAT1, DRD2, and DRD4) were more sensitive to maternal negative reactivity, showing poorer self-control; but these children did not show sensitivity to more positive aspects such as maternal responsiveness (144). Evidence for a differential susceptibility model has been demonstrated in several studies (18–21, 38, 143), while more susceptible children showed sensitivity to both negative and positive effects of the home environment or parenting, in the prediction of several aspects of EF. For example, Sour et al. found that child temperamental surgency moderated the relation between guided learning (i.e., a positive aspect of parenting in which parents assist their child to acquire cognitive skills in the context of problem-solving tasks) and EF; specifically, children who showed high surgency (i.e., high temperamental susceptibility), showed the lowest levels of EF under a low quality of guided learning but had higher EF under a high quality of guided learning. Such a relation was not found among children who showed low levels of surgency (i.e., low temperamental susceptibility) (21).

It should be noted that additional susceptibility factors involved in the development of EF that have been found in these mentioned studies include temperamental reactivity (38), physiological reactivity (19), difficult temperament (143), candidate genes such as the 5-HTTLRP, DAT1, DRD4 (20), and neonatal brain volume (18). All these studies suggested that children with susceptibility factors were more sensitive to both negative and positive aspects of the home environment or parenting; children showed poor EF under less optimal environments but outperformed their non-susceptible peers in supportive environments. Other environmental aspects that children showed sensitivity to, for better and worst, were adversity and low family income (19, 38), maternal behaviors such as sensitivity and the quality of mother-infant interactions (18, 143), and peer affiliation (20).

## Discussion

In this review, we presented the plausible role of sensitivity to the environment in the developmental course of ADHD; we mainly focused on studies that examined sensitivity to positive aspects of parenting and home environment in the development of ADHD and associated deficits in the EF domains. Studies that directly tested the outcome of ADHD symptoms provided evidence to support the general idea of sensitivity to the environment, but the specific pattern of sensitivity was inconsistent between studies. A more consistent pattern of differential susceptibility was found for cognitive functioning in the EF domain, which may be involved in the developmental pathways leading to ADHD.

As mentioned earlier, it should be taken into consideration that the outcome of ADHD symptomatology, compared to the outcome of EF, may not cover the full range from negative outcome to positive outcome (i.e., the lack of symptoms does not necessarily indicate a positive developmental outcome), therefore, it is more complex to test and interpret the specific sensitivity pattern (i.e., diathesis-stress, differential susceptibility, and vantage sensitivity) in the case of ADHD symptoms. Still, despite the different patterns, it seems plausible that sensitivity to the environment exists in the developmental pathways leading directly, or indirectly, through cognitive deficits in the EF domain, to ADHD; and this could help identify children who could benefit most from early intervention programs.

The factors that were found to indicate sensitivity to the environment have been quite consistent across studies. Mainly, variations of candidate genes (e.g., DRD4, DAT1, 5-HTTLRP), aspects of temperament (especially reactivity, inhibitory control, effortful control), parental ADHD symptoms, and neonatal brain volume, were all found to moderate the relation between environmental factors and the developmental outcomes of ADHD and EF.

The parenting and home environment aspects tested in the reviewed papers demonstrated a variety of domains, including the mother’s personality and caregiving behaviors (12, 18, 22, 96, 117, 143), familial conflict, hostility, and home chaos (13, 14, 16), aspects of cognitive stimulation or guided learning (17, 21), socioeconomic status (19, 38), and more. However, since each study focused on one or a small number of environmental factors (as well as susceptibility factors), the question of whether sensitive children are generally more sensitive to all environmental input (i.e., domain-generality) or only to specific environmental input (i.e., domain-specificity) remains open. This important question has been repeatedly raised in the literature (27, 34, 145) but has not been empirically tested until recently (146–149). Belsky et al. (146) found that different children were sensitive to the different environmental attributes of the childcare effect. Specifically, those who showed sensitivity to the effects of child-care quality were not necessarily sensitive to the effects of child-care quantity. Markovitch and Knafo-Noam (148) found that adolescents who perceived themselves as highly sensitive to their parents were less likely to be sensitive to their peers; meaning that sensitivity to one environmental domain was not dependent on sensitivity to another domain. Based on this evidence, they suggested that sensitivity may not be a person-based trait (meaning that a person is more/less sensitive), but rather that this sensitivity may be domain-specific. Furthermore, Sayler et al. (149) found that children who were most and least sensitive to the quality of parenting, showed a similar level of sensitivity to their peers, implying domain-generality. However, this was not the case for all children; some children vary in their sensitivity to different environmental settings. Therefore, instead of just trying to identify who is sensitive and who is less sensitive, we should also try to determine the environmental aspect (or several aspects) that each individual is most sensitive to.

Furthermore, the developmental nature of ADHD, as well as its early onset, may help identify early markers that may foresee its development. The possibility that certain early markers may provide a hallmark for the prediction of a particular outcome in the future, or reflects sensitivity to the environment, raises an important question regarding the specificity of such a prediction. Namely, given that one can properly identify certain markers of susceptibility in early childhood, how informative can this information be to the prediction of a particular outcome and not another? Aspects of temperamental reactivity and self-regulation have been found to predict other developmental outcomes, such as externalizing and internalizing problems (97, 150). Similarly, candidate genes that were found to be related to ADHD were also related to other psychopathologies and therefore they are not considered specific markers of ADHD (72, 75), or specific susceptibility factors, that may be involved in the development of ADHD.

The ability to identify such markers may be improved in several ways; for example, by assessing the combination of different temperament markers instead of focusing only on a specific temperamental domain. Such an approach was used, for example, by Willoughby et al., who found that an elevated level of risk for ADHD was predicted by the temperament profiles of (1) high fear, high anger, and low self-regulation; (2) low fear and low self-regulation; and (3) moderated by anger and moderated activity (98); However, as far as we know, this approach was not used to identify sensitivity to the environment so far. Another factor that may contribute to the identification of children at specific risk for ADHD is the parental history of ADHD or parental ADHD symptomatology (16, 17). ADHD is a highly heritable disorder and children of parents with ADHD are at increased risk of being diagnosed with it themselves (5, 9, 151, 152). Therefore, parental ADHD symptoms may be the first indication that a child may be at risk of developing symptoms of ADHD in the future or manifesting the full disorder. The combination of parental history of ADHD and the child’s temperamental profile could help detect children at such a risk, perhaps even without specific genetic information, and test whether such specific *risk* for ADHD may actually reflect sensitivity to the environment.

In the context of identifying genetic risk, one of the main limitations of the reviewed genetic studies is that the risk for ADHD was mostly evaluated by specific candidate genes (13, 14, 117, 123). ADHD is rarely caused by a single genetic factor; the etiology of ADHD includes the combined effects of many genetic risk factors, each having a very small effect (1). Traditional studies that tested the associations between a small number of polymorphisms and an outcome of interest, without scanning the entire genome, are now considered obsolete (153). It is important to test the idea of sensitivity to the environment in ADHD according to polygenic risk, as it would contribute to strengthening the validity of results from studies that used the candidate genes approach. However, such an examination seems to be problematic in genome-wide association studies, especially in the case of testing differential susceptibility [for detailed information see the review of Zhang and Belsky (154)].

It should also be considered that all studies that were included in this review were correlative. Although environmental factors may moderate the genetic or temperamental risk for ADHD and alter developmental pathways, they may also be passive markers of certain developmental pathways and not active determinants of these pathways (70). Indeed, the correlative nature of these studies does not allow us to infer causality. However, there is evidence to support the idea that some individuals can benefit more from interventions compared to others (155, 156). For example, Bakermans-Kranenburg and van IJzendoorn conducted a meta-analysis of 22 randomized control trials (that included 3,257 participants) and tested the effect of interventions on several developmental outcomes such as internalizing and externalizing problems, cognitive development, and more. They found that intervention effects were much stronger for individuals with genetic susceptibility factors while the effect of interventions was non-significant in the non-susceptible group (155).

To fully test the plausibility of sensitivity to the environment and its practical and therapeutic implications, several aspects should be addressed in future studies. First, potential susceptibility factors should be more broadly assessed; for example, by polygenic risk, several temperamental aspects or temperamental profiles, family history of ADHD—and even the combination of these factors. Second, the assessment of the home environment and parental variables should also be broader; a broader assessment would enable future studies to determine which children are sensitive to which aspects of their environment. Early identification of susceptible children and implementation of interventions that target specific aspects that children may be especially sensitive to, and therefore may benefit most from, may help achieve a better developmental outcome, both in the cognitive domain and in the reduction of their symptomatology. Third, indirect relations should be considered, as cognitive functioning may mediate the relation between the susceptibility factors and the outcome of ADHD symptoms (17). The addressing of these issues could lead to a more customized and efficient approach to preventive interventions.

## Author contributions

TE undertook the review under the supervision of AB. TE wrote the first draft of the manuscript. Both authors developed the initial idea for this review, contributed to the article, and have approved the final manuscript.

## Conflict of interest

The authors declare that the research was conducted in the absence of any commercial or financial relationships that could be construed as a potential conflict of interest.

## Publisher’s note

All claims expressed in this article are solely those of the authors and do not necessarily represent those of their affiliated organizations, or those of the publisher, the editors and the reviewers. Any product that may be evaluated in this article, or claim that may be made by its manufacturer, is not guaranteed or endorsed by the publisher.

## Footnotes

1 This candidate gene is an X-linked gene, located on Xp11.23-11.4; it encodes enzymes that are involved in the metabolism of dopamine, serotonin, and norepinephrine, which are thought to play a role in ADHD (75). Previous studies have focused on the functional 30 base-pair (bp) number of tandem repeat (VNTR) polymorphism; this polymorphism consists of alleles of 2, 3, 3.5, 4, and 5 copies; the 2- and 3-repeat alleles are considered to indicate low activity, and the 3.5- and 4-repeat alleles are considered to indicate high activity (123).

2 In this study, other ADHD symptom domains were not assessed.

## References

[1] Faraone SV Banaschewski T Coghill D Zheng Y Biederman J Bellgrove MA The world federation of ADHD international consensus statement: 208 evidence-based conclusions about the disorder. *Neurosci Biobehav Rev.* (2021) 128:789–818. 10.1016/j.neubiorev.2021.01.022 33549739 PMC8328933

[2] Polanczyk GV Willcutt EG Salum GA Kieling C Rohde LA. ADHD prevalence estimates across three decades: an updated systematic review and meta-regression analysis. *Int J Epidemiol.* (2014) 43:434–42. 10.1093/ije/dyt261 24464188 PMC4817588

[3] Fayyad J Sampson NA Hwang I Adamowski T Aguilar-Gaxiola S Al-Hamzawi A The descriptive epidemiology of DSM-IV adult ADHD in the world health organization world mental health surveys. *Atten Defic Hyperact Disord.* (2017) 9:47–65. 10.1007/s12402-016-0208-3 27866355 PMC5325787

[4] Simon V Czobor P Bálint S Mészáros A Bitter I. Prevalence and correlates of adult attention-deficit hyperactivity disorder: meta-analysis. *Br J Psychiatry.* (2009) 194:204–11. 10.1192/bjp.bp.107.048827 19252145

[5] Larsson H Chang Z D’Onofrio BM Lichtenstein P. The heritability of clinically diagnosed attention-deficit/hyperactivity disorder across the life span. *Psychol Med.* (2014) 44:2223–9. 10.1017/S0033291713002493 24107258 PMC4071160

[6] Faraone SV Larsson H. Genetics of attention deficit hyperactivity disorder. *Mol Psychiatry.* (2019) 24:562–75. 10.1038/s41380-018-0070-0 29892054 PMC6477889

[7] Benzies K Mychasiuk R. Fostering family resiliency: a review of the key protective factors. *Child Fam Soc Work.* (2009) 14:103–14. 10.1111/j.1365-2206.2008.00586.x

[8] Masten AS. Ordinary magic: resilience processes in development. *Am Psychol.* (2001) 56:227–38. 10.1037/0003-066X.56.3.227 11315249

[9] Nikolas MA Burt SA. Genetic and environmental influences on ADHD symptom dimensions of inattention and hyperactivity: a meta-analysis. *J Abnorm Psychol.* (2010) 119:1–17. 10.1037/a0018010 20141238

[10] Nigg JT. Attention-deficit/hyperactivity disorder: endophenotypes, structure, and etiological pathways. *Curr Dir Psychol Sci.* (2010) 19:24–9. 10.1177/0963721409359282

[11] Nikolas MA Klump KL Burt SA. Parental involvement moderates etiological influences on attention deficit hyperactivity disorder behaviors in child twins. *Child Dev.* (2015) 86:224–40. 10.1111/cdev.12296 25263271 PMC4331204

[12] Baptista J Belsky J Mesquita A Soares I. Serotonin transporter polymorphism moderates the effects of caregiver intrusiveness on ADHD symptoms among institutionalized preschoolers. *Eur Child Adolesc Psychiatry.* (2017) 26:303–13. 10.1007/s00787-016-0890-x 27430630

[13] Elmore AL Nigg JT Friderici KH Jernigan K Nikolas MA. Does 5HTTLPR genotype moderate the association of family environment with child attention-deficit hyperactivity disorder symptomatology? *J Clin Child Adolesc Psychol.* (2016) 45:348–60. 10.1080/15374416.2014.979935 25602736 PMC4508252

[14] Martel MM Nikolas M Jernigan K Friderici K Waldman I Nigg JT. The dopamine receptor D4 gene (DRD4) moderates family environmental effects on ADHD. *J Abnorm Child Psychol.* (2011) 39:1–10. 10.1007/s10802-010-9439-5 20644990 PMC4306231

[15] Rioux C Murray J Castellanos-Ryan N Séguin JR Tremblay RE Parent S. Moderation of parenting by inhibitory control in the prediction of the common and unique variance of hyperactivity-impulsivity and inattention. *Dev Psychopathol.* (2020) 32:909–21. 10.1017/S0954579419000774 31409437

[16] Auerbach JG Zilberman-Hayun Y Atzaba-Poria N Berger A. The contribution of maternal ADHD symptomatology, maternal DAT1, and home atmosphere to child ADHD symptomatology at 7 years of age. *J Abnorm Child Psychol.* (2017) 45:415–27. 10.1007/s10802-016-0230-0 27873141

[17] Einziger T Zilberman-Hayun Y Atzaba-Poria N Auerbach JG Berger A. From early risk via cognitive functioning to ADHD phenotype: a longitudinal study of boys at familial risk for ADHD. *Early Child Res Q.* (2021) 57:178–90. 10.1016/j.ecresq.2021.06.003

[18] Nolvi S Rasmussen JM Graham AM Gilmore JH Styner M Fair DA Neonatal brain volume as a marker of differential susceptibility to parenting quality and its association with neurodevelopment across early childhood. *Dev Cogn Neurosci.* (2020) 45:100826. 10.1016/j.dcn.2020.100826 32807730 PMC7393458

[19] Obradović J Portilla XA Ballard PJ. Biological sensitivity to family income: differential effects on early executive functioning. *Child Dev.* (2016) 87:374–84. 10.1111/cdev.12475 26709089

[20] Richards JS Arias Vásquez A van Rooij D van der Meer D Franke B Hoekstra PJ Testing differential susceptibility: plasticity genes, the social environment, and their interplay in adolescent response inhibition. *World J Biol Psychiatry.* (2017) 18:308–21. 10.3109/15622975.2016.1173724 27170266 PMC5435559

[21] Suor JH Sturge-Apple ML Davies PT Jones-Gordils HR. The interplay between parenting and temperament in associations with children’s executive function. *J Fam Psychol.* (2019) 33:841–50. 10.1037/fam0000558 31328944 PMC6776672

[22] Xing S Gao X Liu X Ma Y Wang Z. Maternal personality and child temperamental reactivity: differential susceptibility for child externalizing behavioral problems in China. *Front Psychol.* (2018) 9:1952. 10.3389/fpsyg.2018.01952 30369899 PMC6194149

[23] Belsky J Bakermans-Kranenburg MJ Van IJzendoorn MH. For better and for worse: differential susceptibility to environmental influences. *Curr Dir Psychol Sci.* (2007) 16:300–4. 10.1111/j.1467-8721.2007.00525.x

[24] Bakermans-Kranenburg MJ Van Ijzendoorn MH. Gene-environment interaction of the dopamine D4 receptor (DRD4) and observed maternal insensitivity predicting externalizing behavior in preschoolers. *Dev Psychobiol.* (2006) 48:406–9. 10.1002/dev.20152 16770765

[25] Bakermans-Kranenburg MJ Van Ijzendoorn MH. Differential susceptibility to rearing environment depending on dopamine-related genes: new evidence and a meta-analysis. *Dev Psychopathol.* (2011) 23:39–52. 10.1017/S0954579410000635 21262038

[26] Belsky J. Differential susceptibility to environmental influences. *Int J Child Care Edu Policy.* (2013) 7:15–31. 10.1007/2288-6729-7-2-15

[27] Belsky J Pluess M. Beyond diathesis stress: differential susceptibility to environmental influences. *Psychol Bull.* (2009) 135:885–908. 10.1037/a0017376 19883141

[28] Cho J Kogan SM Brody GH. Genetic moderation of transactional relations between parenting practices and child self-regulation. *J Fam Psychol.* (2016) 30:780–90. 10.1037/fam0000228 27548745 PMC5048522

[29] Rioux C Castellanos-Ryan N Parent S Séguin JR. The interaction between temperament and the family environment in adolescent substance use and externalizing behaviors: support for diathesis–stress or differential susceptibility? *Dev Rev.* (2016) 40:117–50. 10.1016/j.dr.2016.03.003 27413247 PMC4939280

[30] Slagt M Semon Dubas J van Aken MA. Differential susceptibility to parenting in middle childhood: do impulsivity, effortful control and negative emotionality indicate susceptibility or vulnerability? *Infant Child Dev.* (2016) 25:302–24. 10.1002/icd.1929

[31] Van IJzendoorn MH Belsky J Bakermans-Kranenburg MJ. Serotonin transporter genotype 5HTTLPR as a marker of differential susceptibility? A meta-analysis of child and adolescent gene-by-environment studies. *Transl Psychiat.* (2012) 2:e147–147. 10.1038/tp.2012.73 22872162 PMC3432188

[32] Windhorst DA Mileva-Seitz VR Linting M Hofman A Jaddoe VW Verhulst FC Differential susceptibility in a developmental perspective: DRD4 and maternal sensitivity predicting externalizing behavior. *Dev Psychobiol.* (2015) 57:35–49. 10.1002/dev.21257 25251423

[33] Monroe SM Simons AD. Diathesis-stress theories in the context of life stress research: implications for the depressive disorders. *Psychol Bull.* (1991) 110:406–25. 10.1037/0033-2909.110.3.406 1758917

[34] Pluess M Belsky J. Vantage sensitivity: individual differences in response to positive experiences. *Psychol Bull.* (2013) 139:901–16. 10.1037/a0030196 23025924

[35] Pluess M. Individual differences in environmental sensitivity. *Child Dev Perspect.* (2015) 9:138–43. 10.1111/cdep.12120

[36] Boyce WT Ellis BJ. Biological sensitivity to context: I. An evolutionary–developmental theory of the origins and functions of stress reactivity. *Dev Psychopathol.* (2005) 17:271–301. 10.1017/S0954579405050145 16761546

[37] Pluess M Belsky J. Prenatal programming of postnatal plasticity? *Dev Psychopathol.* (2011) 23:29–38. 10.1017/S0954579410000623 21262037

[38] Raver CC Blair C Willoughby M. Poverty as a predictor of 4-year-olds’ executive function: new perspectives on models of differential susceptibility. *Dev Psychol.* (2013) 49:292–304. 10.1037/a0028343 22563675 PMC5460626

[39] Pennington BF McGrath LM Rosenberg J Barnard H Smith SD Willcutt EG Gene X environment interactions in reading disability and attention-deficit/hyperactivity disorder. *Dev Psychol.* (2009) 45:77–89. 10.1037/a0014549 19209992 PMC2743891

[40] Jaekel J Sorg C Breeman L Baumann N Bilgin A Bäuml JG Early regulatory problems and parenting: life-long risk, vulnerability or susceptibility for attention, internalizing and externalizing outcomes? *Eur Child Adolesc Psychiatry.* (2021) 30:1523–31. 10.1007/s00787-020-01632-2 32888096

[41] Stoltz S Beijers R Smeekens S Deković M. Diathesis stress or differential susceptibility? Testing longitudinal associations between parenting, temperament, and children’s problem behavior. *Soc Dev.* (2017) 26:783–96. 10.1111/sode.12237

[42] Arana CC de Pauw SS van IJzendoorn MH de Maat DA Kok R Prinzie P. No differential susceptibility or diathesis stress to parenting in early adolescence: personality facets predicting behaviour problems. *Pers Individ Differ.* (2021) 170:110406. 10.1016/j.paid.2020.110406

[43] Faraone SV Biederman J Mick E. The age-dependent decline of attention deficit hyperactivity disorder: a meta-analysis of follow-up studies. *Psychol Med.* (2006) 36:159–65. 10.1017/S003329170500471X 16420712

[44] Langley K Fowler T Ford T Thapar AK van den Bree M Harold G Adolescent clinical outcomes for young people with attention-deficit hyperactivity disorder. *Br J Psychiatry.* (2010) 196:235–40. 10.1192/bjp.bp.109.066274 20194547

[45] van Lieshout M Luman M Twisk JW van Ewijk H Groenman AP Thissen AJ A 6-year follow-up of a large European cohort of children with attention-deficit/hyperactivity disorder-combined subtype: outcomes in late adolescence and young adulthood. *Eur Child Adolesc Psychiatry.* (2016) 25:1007–17. 10.1007/s00787-016-0820-y 26837866 PMC4990613

[46] Ek U Westerlund J Holmberg K Fernell E. Academic performance of adolescents with ADHD and other behavioural and learning problems - a population-based longitudinal study: academic performance of adolescents with ADHD. *Acta Paediatr.* (2011) 100:402–6. 10.1111/j.1651-2227.2010.02048.x 21054512

[47] Weyandt L DuPaul GJ Verdi G Rossi JS Swentosky AJ Vilardo BS The performance of college students with and without ADHD: neuropsychological, academic, and psychosocial functioning. *J Psychopathol Behav Assess.* (2013) 35:421–35. 10.1007/s10862-013-9351-8 27831696

[48] Kessler RC Adler L Ames M Barkley RA Birnbaum H Greenberg P The prevalence and effects of adult attention deficit/hyperactivity disorder on work performance in a nationally representative sample of workers. *J Occup Environ Med.* (2005) 47:565–72. 10.1097/01.jom.0000166863.33541.39 15951716

[49] Beheshti A Chavanon M-L Christiansen H. Emotion dysregulation in adults with attention deficit hyperactivity disorder: a meta-analysis. *BMC Psychiatry.* (2020) 20:120. 10.1186/s12888-020-2442-7 32164655 PMC7069054

[50] Friedman SR Rapport LJ Lumley M Tzelepis A VanVoorhis A Stettner L Aspects of social and emotional competence in adult attention-deficit/hyperactivity disorder. *Neuropsychology.* (2003) 17:50–8. 10.1037/0894-4105.17.1.50 12597073

[51] Mannuzza S Klein RG. Long-term prognosis in attention-deficit/hyperactivity disorder. *Child Adolesc Psychiatr Clin N Am.* (2000) 9:711–26. 10.1016/S1056-4993(18)30114-7 10944664

[52] Van der Oord S Van der Meulen EM Prins PJM Oosterlaan J Buitelaar JK Emmelkamp PMG. A psychometric evaluation of the social skills rating system in children with attention deficit hyperactivity disorder. *Behav Res Ther.* (2005) 43:733–46. 10.1016/j.brat.2004.06.004 15890166

[53] Ros R Graziano PA. Social functioning in children with or at risk for attention deficit/hyperactivity disorder: a meta-analytic review. *J Clin Child Adolesc Psychol.* (2018) 47:213–35. 10.1080/15374416.2016.1266644 28128989

[54] Pollak Y Dekkers TJ Shoham R Huizenga HM. Risk-Taking behavior in attention deficit/hyperactivity disorder (ADHD): a review of potential underlying mechanisms and of interventions. *Curr Psychiatry Rep.* (2019) 21:33. 10.1007/s11920-019-1019-y 30903380

[55] Capusan AJ Bendtsen P Marteinsdottir I Larsson H. Comorbidity of adult ADHD and its subtypes with substance use disorder in a large population-based epidemiological study. *J Atten Disord.* (2019) 23:1416–26. 10.1177/1087054715626511 26838558

[56] Charach A Yeung E Climans T Lillie E. Childhood attention-deficit/hyperactivity disorder and future substance use disorders: comparative meta-analyses. *J Am Acad Child Adolesc Psychiatry.* (2011) 50:9–21. 10.1016/j.jaac.2010.09.019 21156266

[57] Mannuzza S Klein RG Moulton JL. Lifetime criminality among boys with attention deficit hyperactivity disorder: a prospective follow-up study into adulthood using official arrest records. *Psychiatry Res.* (2008) 160:237–46. 10.1016/j.psychres.2007.11.003 18707766 PMC2581455

[58] Mohr-Jensen C Bisgaard CM Boldsen SK Steinhausen H-C. Attention-deficit/hyperactivity disorder in childhood and adolescence and the risk of crime in young adulthood in a Danish nationwide study. *J Am Acad Child Adolesc Psychiatry.* (2019) 58:443–52. 10.1016/j.jaac.2018.11.016 30768385

[59] Chang Z Lichtenstein P D’Onofrio BM Sjölander A Larsson H. Serious transport accidents in adults with attention-deficit/hyperactivity disorder and the effect of medication: a population-based study. *JAMA Psychiatry.* (2014) 71:319–25. 10.1001/jamapsychiatry.2013.4174 24477798 PMC3949159

[60] Theule J Hurl KE Cheung K Ward M Henrikson B. Exploring the relationships between problem gambling and ADHD: a meta-analysis. *J Atten Disord.* (2019) 23:1427–37. 10.1177/1087054715626512 26832122

[61] American Psychiatric Association. *Diagnostic and Statistical Manual of Mental Disorders (DSM-5§).* Washington, DC: American Psychiatric Pub (2013). 10.1176/appi.books.9780890425596

[62] Cuffe SP Visser SN Holbrook JR Danielson ML Geryk LL Wolraich ML ADHD and psychiatric comorbidity: functional outcomes in a school-based sample of children. *J Atten Disord.* (2020) 24:1345–54. 10.1177/1087054715613437 26610741 PMC4879105

[63] Riglin L Leppert B Dardani C Thapar AK Rice F O’Donovan MC ADHD and depression: investigating a causal explanation. *Psychol Med.* (2020) 51:1890–7. 10.1017/S0033291720000665 32249726 PMC8381237

[64] Treur JL Demontis D Smith GD Sallis H Richardson TG Wiers RW Investigating causality between liability to ADHD and substance use, and liability to substance use and ADHD risk, using mendelian randomization. *Addict Biol.* (2021) 26:e12849. 10.1111/adb.12849 31733098 PMC7228854

[65] Doshi JA Hodgkins P Kahle J Sikirica V Cangelosi MJ Setyawan J Economic impact of childhood and adult attention-deficit/hyperactivity disorder in the United States. *J Am Acad Child Adolesc Psychiatry.* (2012) 51:990–1002. 10.1016/j.jaac.2012.07.008 23021476

[66] Pelham WE Foster EM Robb JA. The economic impact of attention-deficit/hyperactivity disorder in children and adolescents. *J Pediatr Psychol.* (2007) 32:711–27. 10.1093/jpepsy/jsm022 17556402

[67] Barkley RA. Recent longitudinal studies of childhood attention-deficit/hyperactivity disorder: important themes and questions for further research. *J Abnorm Psychol.* (2016) 125:248–55. 10.1037/abn0000125 26854509

[68] Rueda MR Rothbart MK McCandliss BD Saccomanno L Posner MI. Training, maturation, and genetic influences on the development of executive attention. *Proc Natl Acad Sci USA.* (2005) 102:14931–6. 10.1073/pnas.0506897102 16192352 PMC1253585

[69] Vukšić M Radoš M Kostović I. Structural basis of developmental plasticity in the corticostriatal system. *Coll Antropo.* (2008) 32(Suppl. 1):155–9. 18405076

[70] Sonuga-Barke EJS Halperin JM. Developmental phenotypes and causal pathways in attention deficit/hyperactivity disorder: potential targets for early intervention? Developmental phenotypes and causal pathways in ADHD. *J Child Psychol Psychiatry.* (2010) 51:368–89. 10.1111/j.1469-7610.2009.02195.x 20015192

[71] Faraone SV Bonvicini C Scassellati C. Biomarkers in the diagnosis of ADHD–promising directions. *Curr Psychiatry Rep.* (2014) 16:497. 10.1007/s11920-014-0497-1 25298126

[72] Akutagava-Martins GC Salatino-Oliveira A Kieling CC Rohde LA Hutz MH. Genetics of attention-deficit/hyperactivity disorder: current findings and future directions. *Expert Rev Neurother.* (2013) 13:435–45. 10.1586/ern.13.30 23545057

[73] Pettersson E Lichtenstein P Larsson H Song J Deficit A Agrawal A Genetic influences on eight psychiatric disorders based on family data of 4 408 646 full and half-siblings, and genetic data of 333 748 cases and controls. *Psychol Med.* (2019) 49:1166–73. 30221610 10.1017/S0033291718002039 PMC6421104

[74] Gizer IR Ficks C Waldman ID. Candidate gene studies of ADHD: a meta-analytic review. *Hum Genet.* (2009) 126:51–90. 10.1007/s00439-009-0694-x 19506906

[75] Thapar A Cooper M Eyre O Langley K. Practitioner review: what have we learnt about the causes of ADHD? *J Child Psychol Psychiatry.* (2013) 54:3–16. 10.1111/j.1469-7610.2012.02611.x 22963644 PMC3572580

[76] El-Faddagh M Laucht M Maras A Vöhringer L Schmidt MH. Association of dopamine D4 receptor (DRD4) gene with attention-deficit/hyperactivity disorder (ADHD) in a high-risk community sample: a longitudinal study from birth to 11 years of age. *J Neural Transm.* (2004) 111:883–9. 10.1007/s00702-003-0054-2 15206004

[77] Franke B Vasquez AA Johansson S Hoogman M Romanos J Boreatti-Hümmer A Multicenter analysis of the SLC6A3/DAT1 VNTR haplotype in persistent ADHD suggests differential involvement of the gene in childhood and persistent ADHD. *Neuropsychopharmacol.* (2010) 35:656–64. 10.1038/npp.2009.170 19890261 PMC3055604

[78] Manor I Eisenberg J Tyano S Sever Y Cohen H Ebstein RP Family-based association study of the serotonin transporter promoter region polymorphism (5-HTTLPR) in attention deficit hyperactivity disorder. *Am J Med Genet.* (2001) 105:91–5. 10.1002/1096-8628(20010108)105:1<91::AID-AJMG1069>3.0.CO;2-V 11425009

[79] Wargelius H-L Malmberg K Larsson J-O Oreland L. Associations of MAOA-VNTR or 5HTT-LPR alleles with attention-deficit hyperactivity disorder symptoms are moderated by platelet monoamine oxidase B activity. *Psychiatr Genet.* (2012) 22:42–5. 10.1097/YPG.0b013e328347c1ab 21610556

[80] Demontis D Walters RK Martin J Mattheisen M Als TD Agerbo E Discovery of the first genome-wide significant risk loci for attention deficit/hyperactivity disorder. *Nat Genet.* (2019) 51:63–75. 30478444 10.1038/s41588-018-0269-7 PMC6481311

[81] Taylor MJ Martin J Lu Y Brikell I Lundström S Larsson H Association of genetic risk factors for psychiatric disorders and traits of these disorders in a Swedish population twin sample. *JAMA Psychiatry.* (2019) 76:280–9. 10.1001/jamapsychiatry.2018.3652 30566181 PMC6439816

[82] Lee PH Anttila V Won H Grünblatt E Walitza S. Genome wide meta-analysis identifies genomic relationships, novel loci, and pleiotropic mechanisms across eight psychiatric disorders. *bioRxiv.* (2019) 179:1469–82. 31835028 10.1016/j.cell.2019.11.020 PMC7077032

[83] Goldsmith HH Buss KA Lemery KS. Toddler and childhood temperament: expanded content, stronger genetic evidence, new evidence for the importance of environment. *Dev Psychol.* (1997) 33:891–905. 10.1037/0012-1649.33.6.891 9383612

[84] Rothbart MK Derryberry D. Development of individual differences in temperament. In: Lamb MB Brown AL editors. *Advances in Developmental Psychology.* Hillsdale, NJ: Lawrence Erlbaum Associates (1981). p. 37–86.

[85] Rothbart MK Ahadi SA Evans DE. Temperament and personality: origins and outcomes. *J Pers Soc Psychol.* (2000) 78:122–35. 10.1037/0022-3514.78.1.122 10653510

[86] Posner MI Rothbart MK. Developing mechanisms of self-regulation. *Dev Psychopathol.* (2000) 12:427–41. 10.1017/S0954579400003096 11014746

[87] Rothbart MK Ahadi SA Hershey KL Fisher P. Investigations of temperament at three to seven years: the Children’s behavior questionnaire. *Child Dev.* (2001) 72:1394–408. 10.1111/1467-8624.00355 11699677

[88] Martel MM Nigg JT. Child ADHD and personality/temperament traits of reactive and effortful control, resiliency, and emotionality. *J Child Psychol Psychiatry.* (2006) 47:1175–83. 10.1111/j.1469-7610.2006.01629.x 17076757

[89] Nigg JT Goldsmith HH Sachek J. Temperament and attention deficit hyperactivity disorder: the development of a multiple pathway model. *J Clin Child Adolesc Psychol.* (2004) 33:42–53. 10.1207/S15374424JCCP3301_5 15028540

[90] Sonuga-Barke EJS Auerbach JG Campbell SB Daley D Thompson M. Varieties of preschool hyperactivity: multiple pathways from risk to disorder. *Dev Sci.* (2005) 8:141–50. 10.1111/j.1467-7687.2005.00401.x 15720372

[91] De Pauw SS Mervielde I. The role of temperament and personality in problem behaviors of children with ADHD. *J Abnorm Child Psychol.* (2011) 39:277–91. 10.1007/s10802-010-9459-1 20862537

[92] Einziger T Levi L Zilberman-Hayun Y Auerbach JG Atzaba-Poria N Arbelle S Predicting ADHD symptoms in adolescence from early childhood temperament traits. *J Abnorm Child Psychol.* (2018) 46:265–76. 10.1007/s10802-017-0287-4 28317068

[93] Herzhoff K Tackett JL Martel MM. A dispositional trait framework elucidates differences between interview and questionnaire measurement of childhood attention problems. *Psychol Assess.* (2013) 25:1079–90. 10.1037/a0033008 23730832

[94] Martel MM Nigg JT Von Eye A. How do trait dimensions map onto ADHD symptom domains? *J Abnorm Child Psychol.* (2009) 37:337–48. 10.1007/s10802-008-9255-3 18668361 PMC2818785

[95] Martel MM Gremillion ML Roberts BA Zastrow BL Tackett JL. Longitudinal prediction of the one-year course of preschool ADHD symptoms: implications for models of temperament–ADHD associations. *Pers Individ Diff.* (2014) 64:58–61. 10.1016/j.paid.2014.02.018 25598568 PMC4295518

[96] Miller NV Degnan KA Hane AA Fox NA Chronis-Tuscano A. Infant temperament reactivity and early maternal caregiving: independent and interactive links to later childhood attention-deficit/hyperactivity disorder symptoms. *J Child Psychol Psychiatr.* (2019) 60:43–53. 10.1111/jcpp.12934 29889314 PMC6289898

[97] Wichstrøm L Penelo E Rensvik Viddal K de la Osa N Ezpeleta L. Explaining the relationship between temperament and symptoms of psychiatric disorders from preschool to middle childhood: hybrid fixed and random effects models of Norwegian and Spanish children. *J Child Psychol Psychiatry.* (2018) 59:285–95. 10.1111/jcpp.12772 28671298

[98] Willoughby MT Gottfredson NC Stifter CA The Family Life Project Investigators. Observed temperament from ages 6 to 36 months predicts parent- and teacher-reported attention-deficit/hyperactivity disorder symptoms in first grade. *Dev Psychopathol.* (2017) 29:107–20. 10.1017/S0954579415001236 26751219

[99] Goldsmith HH Lemery KS Essex MJ. Temperament as a liability factor for childhood behavioral disorders: the concept of liability. In: DiLalla LF editor. *Behavior Genetic Principles: Perspectives in Development, Personality, and Psychopathology.* Washington, DC: American Psychological Association (2004). p. 19–39. 10.1037/10684-002

[100] Bussing R Gary FA Mason DM Leon CE Sinha K Garvan CW. Child temperament, ADHD, and caregiver strain: exploring relationships in an epidemiological sample. *J Am Acad Child Adolesc Psychiatry.* (2003) 42:184–92. 10.1097/00004583-200302000-00012 12544178

[101] Hare MM Graziano PA. Treatment response among preschoolers with disruptive behavior disorders: the role of temperament and parenting. *J Clin Child Adolesc Psychol.* (2021) 50:950–65. 10.1080/15374416.2020.1846540 33275456 PMC8175459

[102] Thorell LB Wåhlstedt C. Executive functioning deficits in relation to symptoms of ADHD and/or ODD in preschool children. *Inf Child Dev.* (2006) 15:503–18. 10.1002/icd.475

[103] Martel MM Elkins AR Eng AG Goh PK Bansal PS Smith-Thomas TE Longitudinal temperament pathways to ADHD between childhood and adolescence. *Res Child Adolesc Psychopathol.* (2022):1–12. 10.1007/s10802-022-00902-8 35102487 PMC9680910

[104] Banerjee TD Middleton F Faraone SV. Environmental risk factors for attention-deficit hyperactivity disorder. *Acta Paediatr.* (2007) 96:1269–74. 10.1111/j.1651-2227.2007.00430.x 17718779

[105] Hinshaw SP. Attention deficit hyperactivity disorder (ADHD): controversy, developmental mechanisms, and multiple levels of analysis. *Annu Rev Clin Psychol.* (2018) 14:291–316. 10.1146/annurev-clinpsy-050817-084917 29220204

[106] Sciberras E Mulraney M Silva D Coghill D. Prenatal risk factors and the etiology of ADHD—review of existing evidence. *Curr Psychiatry Rep.* (2017) 19:1. 10.1007/s11920-017-0753-2 28091799

[107] Nigg JT Nikolas M Burt SA. Measured gene-by-environment interaction in relation to attention-deficit/hyperactivity disorder. *J Am Acad Child Adolesc Psychiatry.* (2010) 49:863–73. 10.1016/j.jaac.2010.01.025 20732623 PMC2928573

[108] Breaux RP Harvey EA. A longitudinal study of the relation between family functioning and preschool ADHD symptoms. *J Clin Child Adolesc Psychol.* (2019) 48:749–64. 10.1080/15374416.2018.1437737 29578799 PMC12188987

[109] Klein MR Lengua LJ Thompson SF Moran L Ruberry EJ Kiff C Bidirectional relations between temperament and parenting predicting preschool-age children’s adjustment. *J Clin Child Adolesc Psychol.* (2018) 47:S113–26. 10.1080/15374416.2016.1169537 27399174 PMC6175662

[110] Wittig SM Rodriguez CM. Emerging behavior problems: bidirectional relations between maternal and paternal parenting styles with infant temperament. *Dev Psychol.* (2019) 55:1199–210. 10.1037/dev0000707 30742467 PMC6533133

[111] Barkley RA Fischer M Edelbrock C Smallish L. The adolescent outcome of hyperactive children diagnosed by research criteria—III. Mother–child interactions, family conflicts and maternal psychopathology. *J Child Psychol Psychiatry.* (1991) 32:233–55. 10.1111/j.1469-7610.1991.tb00304.x 2033106

[112] Zvara BJ Sheppard KW Cox M. Bidirectional effects between parenting sensitivity and child behavior: a cross-lagged analysis across middle childhood and adolescence. *J Fam Psychol.* (2018) 32:484–95. 10.1037/fam0000372 29697996 PMC7466908

[113] Einziger T Zilberman-Hayun Y Atzaba-Poria N Auerbach JG Berger A. How important is early home environment in the prediction of attention-deficit hyperactivity disorder in adolescence? The protective role of early cognitive stimulation. *Inf Child Dev.* (2019) 28:e2138. 10.1002/icd.2138

[114] Hawes DJ Dadds MR Frost AD Russell A. Parenting practices and prospective levels of hyperactivity/inattention across early-and middle-childhood. *J Psychopathol and Behav Assess.* (2013) 35:273–82. 10.1007/s10862-013-9341-x

[115] Lai WW O’Mahony M Mulligan A. The home observation measure of the environment is associated with symptoms of ADHD and oppositionality in a CAMHS sample. *Clin Child Psychol Psychiatry.* (2018) 23:503–13. 10.1177/1359104517740712 29262691

[116] Mulligan A Anney R Butler L O’Regan M Richardson T Tulewicz EM Home environment: association with hyperactivity/impulsivity in children with ADHD and their non-ADHD siblings. *Child Care Health Dev.* (2013) 39:202–12. 10.1111/j.1365-2214.2011.01345.x 22168816 PMC3307872

[117] Li JJ Lee SS. Interaction of dopamine transporter gene and observed parenting behaviors on attention-deficit/hyperactivity disorder: a structural equation modeling approach. *J Clin Child Adolesc Psychol.* (2013) 42:174–86. 10.1080/15374416.2012.736355 23153115 PMC3586755

[118] Laucht M Skowronek MH Becker K Schmidt MH Esser G Schulze TG Interacting effects of the dopamine transporter gene and psychosocial adversity on attention-deficit/hyperactivity disorder symptoms among 15-year-olds from a high-risk community sample. *Arch Gen Psychiatry.* (2007) 64:585–90. 10.1001/archpsyc.64.5.585 17485610

[119] Retz W Freitag CM Retz-Junginger P Wenzler D Schneider M Kissling C A functional serotonin transporter promoter gene polymorphism increases ADHD symptoms in delinquents: interaction with adverse childhood environment. *Psychiatry Res.* (2008) 158:123–31. 10.1016/j.psychres.2007.05.004 18155777

[120] Waldman ID. Gene–environment interactions reexamined: does mother’s marital stability interact with the dopamine receptor D2 gene in the etiology of childhood attention-deficit/hyperactivity disorder? *Dev Psychopathol.* (2007) 19:1117–28. 10.1017/S0954579407000570 17931438

[121] Lasky-Su J Faraone SV Lange C Tsuang MT Doyle AE Smoller JW A study of how socioeconomic status moderates the relationship between SNPs encompassing BDNF and ADHD symptom counts in ADHD families. *Behav Genet.* (2007) 37:487–97. 10.1007/s10519-006-9136-x 17216343

[122] Rosenberg J Pennington BF Willcutt EG Olson RK. Gene by environment interactions influencing reading disability and the inattentive symptom dimension of attention deficit/hyperactivity disorder. *J Child Psychol Psychiatry.* (2012) 53:243–51. 10.1111/j.1469-7610.2011.02452.x 21884522 PMC3235245

[123] Li JJ Lee SS. Association of positive and negative parenting behavior with childhood ADHD: interactions with offspring monoamine oxidase A (MAO-A) genotype. *J Abnorm Child Psychol.* (2012) 40:165–75. 10.1007/s10802-011-9553-z 21826446

[124] Willcutt EG. The prevalence of DSM-IV attention-deficit/hyperactivity disorder: a meta-analytic review. *Neurotherapeutics.* (2012) 9:490–9. 10.1007/s13311-012-0135-8 22976615 PMC3441936

[125] Castellanos FX Sonuga-Barke EJS Milham MP Tannock R. Characterizing cognition in ADHD: beyond executive dysfunction. *Trends Cogn Sci.* (2006) 10:117–23. 10.1016/j.tics.2006.01.011 16460990

[126] Wåhlstedt C Thorell LB Bohlin G. Heterogeneity in ADHD: neuropsychological pathways, comorbidity and symptom domains. *J Abnorm Child Psychol.* (2009) 37:551–64. 10.1007/s10802-008-9286-9 19016322

[127] Kofler MJ Rapport MD Sarver DE Raiker JS Orban SA Friedman LM Reaction time variability in ADHD: a meta-analytic review of 319 studies. *Clin Psychol Rev.* (2013) 33:795–811. 10.1016/j.cpr.2013.06.001 23872284

[128] Doyle AE Willcutt EG Seidman LJ Biederman J Chouinard V-A Silva J Attention-deficit/hyperactivity disorder endophenotypes. *Biol Psychiatry.* (2005) 57:1324–35. 10.1016/j.biopsych.2005.03.015 15950005

[129] Rommelse NNJ Altink ME Martin NC Buschgens CJ Faraone SV Buitelaar JK Relationship between endophenotype and phenotype in ADHD. *Behav Brain Funct.* (2008) 4:4. 10.1186/1744-9081-4-4 18234079 PMC2267799

[130] Gottesman II Gould TD. The endophenotype concept in psychiatry: etymology and strategic intentions. *Am J Psychiatry.* (2003) 160:636–45. 10.1176/appi.ajp.160.4.636 12668349

[131] Kuntsi J Neale BM Chen W Faraone SV Asherson P. The IMAGE project: methodological issues for the molecular genetic analysis of ADHD. *Behav Brain Funct.* (2006) 2:27. 10.1186/1744-9081-2-27 16887023 PMC1559631

[132] Gau SS-F Shang C-Y. Executive functions as endophenotypes in ADHD: evidence from the Cambridge neuropsychological test battery (CANTAB). *J Child Psychol Psychiatry.* (2010) 51:838–49. 10.1111/j.1469-7610.2010.02215.x 20085608

[133] Lin Y-J Gau SS-F. Developmental changes of neuropsychological functioning in individuals with and without childhood ADHD from early adolescence to young adulthood: a 7-year follow-up study. *Psychol Med.* (2019) 49:940–51. 10.1017/S0033291718001599 29941053

[134] Willcutt EG Doyle AE Nigg JT Faraone SV Pennington BF. Validity of the executive function theory of attention-deficit/hyperactivity disorder: a meta-analytic review. *Biol Psychiatry.* (2005) 57:1336–46. 10.1016/j.biopsych.2005.02.006 15950006

[135] Garon N Bryson SE Smith IM. Executive function in preschoolers: a review using an integrative framework. *Psychol Bull.* (2008) 134:31–60. 10.1037/0033-2909.134.1.31 18193994

[136] Welsh MC. Developmental and clinical variations in executive functions. In: Molfese DL Molfese VJ editors. *Developmental Variations in Learning: Applications to Social, Executive Function, Language, and Reading Skills.* Hillsdale, NJ: Lawrence Erlbaum Associates Publishers (2002). p. 139–85.

[137] Almasy L Blangero J. Endophenotypes as quantitative risk factors for psychiatric disease: rationale and study design. *Am J Med Genet.* (2001) 105:42–4. 10.1002/1096-8628(20010108)105:1<42::AID-AJMG1055>3.0.CO;2-9 11424994

[138] Overbeek G. Parenting intervention effects on children’s externalizing behavior: the moderating role of genotype and temperament. *Curr Opin Psychol.* (2017) 15:143–8. 10.1016/j.copsyc.2017.02.025 28813255

[139] Retz W Stieglitz R-D Corbisiero S Retz-Junginger P Rösler M. Emotional dysregulation in adult ADHD: what is the empirical evidence? *Expert Rev Neurother.* (2012) 12:1241–51. 10.1586/ern.12.109 23082740

[140] Friedrich A Moning J Weiss J Schlarb AA. The effects of parental ADHD symptoms on parenting behaviors. *Health.* (2017) 9:1054–74. 10.4236/health.2017.97077

[141] Johnston C Mash EJ Miller N Ninowski JE. Parenting in adults with attention-deficit/hyperactivity disorder (ADHD). *Clin Psychol Rev.* (2012) 32:215–28. 10.1016/j.cpr.2012.01.007 22459785 PMC4838457

[142] Mazursky-Horowitz H Felton JW MacPherson L Ehrlich KB Cassidy J Lejuez CW Maternal emotion regulation mediates the association between adult attention-deficit/hyperactivity disorder symptoms and parenting. *J Abnorm Child Psychol.* (2015) 43:121–31. 10.1007/s10802-014-9894-5 24928735 PMC9358793

[143] Rochette É Bernier A. Parenting and preschoolers’ executive functioning: a case of differential susceptibility? *Int J Behav Dev.* (2016) 40:151–61. 10.1177/0165025414557370

[144] Vrantsidis DM Clark CA Volk A Wakschlag LS Espy KA Wiebe SA. Exploring the interplay of dopaminergic genotype and parental behavior in relation to executive function in early childhood. *Dev Psychopathol.* (2021):1–12. 10.1017/S0954579421001061 34779374 PMC9107528

[145] Ellis BJ Boyce WT Belsky J Bakermans-Kranenburg MJ Van IJzendoorn MH. Differential susceptibility to the environment: an evolutionary–neurodevelopmental theory. *Dev Psychopathol.* (2011) 23:7–28. 10.1017/S0954579410000611 21262036

[146] Belsky J Zhang X Sayler K. Differential susceptibility 2.0: are the same children affected by different experiences and exposures? *Dev Psychopathol.* (2021):1–9. 10.1017/S0954579420002205 33634774

[147] Markovitch N Kirkpatrick RM Knafo-Noam A. Are different individuals sensitive to different environments? Individual differences in sensitivity to the effects of the parent, peer and school environment on externalizing behavior and its genetic and environmental etiology. *Behav Genet.* (2021) 51:492–511. 10.1007/s10519-021-10075-7 34195925

[148] Markovitch N Knafo-Noam A. Sensitivity, but to which environment? Individual differences in sensitivity to parents and peers show domain-specific patterns and a negative genetic correlation. *Dev Sci.* (2021) 24:e13136. 10.1111/desc.13136 34155726

[149] Sayler K Zhang X Steinberg L Belsky J. Parenting, peers and psychosocial adjustment: are the same—or different—children affected by each? *J Youth Adolesc.* (2022) 51:443–57. 10.1007/s10964-022-01574-9 35076830

[150] Fosco WD Hawk LW Jr. Colder CR Meisel SN Lengua LJ. The development of inhibitory control in adolescence and prospective relations with delinquency. *J Adolesc.* (2019) 76:37–47. 10.1016/j.adolescence.2019.08.008 31442813 PMC6803097

[151] Biederman J Faraone SV Mick E Spencer T Wilens T Kiely K High risk for attention deficit hyperactivity disorder among children of parents with childhood onset of the disorder: a pilot study. *Am J Psychiatry.* (1995) 152:431–5. 10.1176/ajp.152.3.431 7864271

[152] Minde K Eakin L Hechtman L Ochs E Bouffard R Greenfield B The psychosocial functioning of children and spouses of adults with ADHD. *J Child Psychol Psychiatry.* (2003) 44:637–46. 10.1111/1469-7610.00150 12751853

[153] Duncan LE Ostacher M Ballon J. How genome-wide association studies (GWAS) made traditional candidate gene studies obsolete. *Neuropsychopharmacol.* (2019) 44:1518–23. 10.1038/s41386-019-0389-5 30982060 PMC6785091

[154] Zhang X Belsky J. Three phases of Gene x Environment interaction research: theoretical assumptions underlying gene selection. *Dev Psychopathol.* (2022) 34:295–306. 10.1017/S0954579420000966 32880244

[155] Bakermans-Kranenburg MJ Van Ijzendoorn MH. The hidden efficacy of interventions: genes X environment experiments from a differential susceptibility perspective. *Ann Rev Psychol.* (2015) 66:381–409. 10.1146/annurev-psych-010814-015407 25148854

[156] de Villiers B Lionetti F Pluess M. Vantage sensitivity: a framework for individual differences in response to psychological intervention. *Soc Psychiatry Psychiatr Epidemiol.* (2018) 53:545–54. 10.1007/s00127-017-1471-0 29302707 PMC5959990

